# Reoccurrence of West Nile virus lineage 1 after 2-year decline: first equine outbreak in Campania region

**DOI:** 10.3389/fvets.2023.1314738

**Published:** 2023-11-30

**Authors:** Claudio de Martinis, Lorena Cardillo, Federica Pesce, Maurizio Viscardi, Loredana Cozzolino, Rubina Paradiso, Stefania Cavallo, Matteo De Ascentis, Maria Goffredo, Federica Monaco, Giovanni Savini, Francescantonio D’Orilia, Renato Pinto, Giovanna Fusco

**Affiliations:** ^1^Department of Animal Health, Istituto Zooprofilattico Sperimentale del Mezzogiorno, Portici, Italy; ^2^Department of Epidemiologic and Biostatistics Regional Observatory (OREB), Istituto Zooprofilattico Sperimentale del Mezzogiorno, Portici, Italy; ^3^Istituto Zooprofilattico Sperimentale di Abruzzo e Molise, Teramo, Italy; ^4^Centro di riferimento regionale Sanità Animale (C.Re.San.), Nocera, Italy; ^5^U.O.D. Prevenzione e sanità pubblica veterinaria, Regione Campania, Napoli, Italy

**Keywords:** West Nile virus, lineage 1, horse, whole genome sequencing, phylogenetic analysis

## Abstract

West Nile virus (WNV) is the most widespread arbovirus worldwide, responsible for severe neurological symptoms in humans as well as in horses and birds. The main reservoir and amplifier of the virus are birds, and migratory birds seem to have a key role in the introduction and spread of WNV during their migratory routes. WNV lineage 1 (L1) has been missing in Italy for almost 10 years, only to reappear in 2020 in two dead raptor birds in southern Italy. The present study reports the first equine outbreak in the Campania region. A 7-year-old horse died because of worsening neurological signs and underwent necropsy and biomolecular analyses. WNV-L1 was detected by real-time RT-PCR in the heart, brain, gut, liver, and spleen. Next Generation Sequence and phylogenetic analysis revealed that the strain responsible for the outbreak showed a nucleotide identity of over 98% with the strain found in *Accipiter gentilis* 2 years earlier in the same area, belonging to the WNV-L1 Western-Mediterranean sub-cluster. These results underline that WNV-L1, after reintroduction in 2020, has probably silently circulated during a 2-year eclipse, with no positive sample revealed by both serological and biomolecular examinations in horses, birds, and mosquitoes. The climate changes that have occurred in the last decades are evolving the epidemiology of WNV, with introductions or re-introductions of the virus in areas that were previously considered low risk. Thereby, the virus may easily amplify and establish itself to reappear with sporadic evident cases in susceptible hosts after several months or even years.

## 1 Introduction

West Nile virus (WNV) is a zoonotic enveloped single-stranded RNA agent that belongs to the *Flaviviridae* family and is transmitted by mosquitoes mainly of the genus *Culex*. This virus is included in the Japanese encephalitis virus serocomplex, along with the Usutu virus, the Murray Valley encephalitis virus, the Stratford virus, the Alfui virus, the Kunjin virus, and the Saint Louis encephalitis virus (1). Although isolated for the first time in 1937 in a febrile woman in the West Nile Province of Uganda (2), it was considered a disease of secondary importance in humans for a long time, acquiring growing concern only in the last two decades (3). Indeed, at present, WNV is considered the most widespread arbovirus, with increasing cases of severe diseases in the affected species, including humans (4, 5). In immunocompetent individuals, the infection is generally asymptomatic or with mild flu-like symptoms, known as West Nile Fever, while children, the elderly, and immunosuppressed individuals could experience a more severe disease. In these cases, the infection can lead to neuro-invasive forms, characterised by meningitis, encephalitis, and acute flaccid paralysis/poliomyelitis, which in one-third of cases can lead to long-term or permanent damage (3, 5).

WNV can infect a plethora of animals, such as birds, horses, sheep, cats, rodents, reptiles, Indian elephants, Indian rhinoceros, ring-tailed lemurs, red pandas, snow leopards, and babirusas (2, 3, 6–9). Many species of birds can act as the main reservoir and amplifier hosts. In these species, WNV infection can cause a high level of viraemia long enough to permit mosquito infection (10). Furthermore, certain raptor and corvid species develop clinical signs that lead to fatal outcomes.

Horses, instead, as well as humans, are dead-end hosts. In this species, the infection is usually asymptomatic, while in 20% of cases, clinical and neurological signs may develop, which can range from fever and mild ataxia to total recumbence, leading to a mortality rate of 30–40% in unvaccinated animals (4, 11).

Currently, strains of WNV are phylogenetically divided into eight distinct lineages, of which 1 (L1) and 2 (L2) are responsible for severe disease in humans and horses (12). Nevertheless, several factors related to the pathogen, hosts, and environment have been identified to influence the virulence of WNV (13). Site-specific mutations have been proven to be the main factor affecting WNV virulence (14). In particular, the NS3 helicase domain is considered a possible virulence determinant, as it is involved in the inhibition of the type I interferon response (15, 16). This mutable strain virulence underlies the different severity of the outbreaks (14).

Recently, WNV has emerged in numerous geographic areas, even becoming endemic in several countries. Major epidemics have been reported globally (5, 17). In 2022, a peak of contagion was reported in Europe. According to the European Centre for Disease Prevention and Control (ECDC), the 2022 epidemic peak recorded the highest number of locally acquired cases since 2018. Italy was one of the most affected countries (18), reporting 588 confirmed human cases of WND, 50% of which developed the neuro-invasive form with 61 deaths (19).

The introduction of the West Nile virus in Italy dates back to 1998 in the Tuscany region during an equine outbreak (20) caused by WNV lineage 1. Thereafter, no further cases were detected until 2008, when, in the area surrounding the Po River Delta, WNV was found in mosquitoes, birds, and humans (21). Up to 2011, the viral circulation in the country was due to strains belonging to L1, becoming endemic in northern regions and spreading to the south (10). After its first evidence in north-eastern Italy in 2011, Eastern European WNV lineage 2 rapidly spread in the whole country, completely overtaking the previously circulating L1 (1). Nevertheless, in 2020, two raptor birds, a kestrel (*Falco tinnunculus*) and a goshawk (*Accipiter gentilis*), were found WNV-L1 positive in the Campania region (10). During the current 2022–2023 vector season, the WNV epidemiological scenario has increased its complexity due to an intense co-circulation of both lineages in many Italian regions (19, 22).

In the whole country, the national surveillance plan for arbovirosis (PNA 2020–2025) aims to monitor WNV circulation on resident birds of targeted species and on dead wild birds, as well as on mosquitoes, equids, and humans. According to this plan, Italian regions are risk-based classified, identifying three different classes: high-risk areas where the virus is currently circulating or has circulated during the previous 5 years, thus outbreaks are frequently observed; low-risk areas where the virus has sporadically circulated in the past or the virus has never been detected, but eco-climatic conditions may be favourable to virus circulation; and minimum-risk areas where the virus has never been detected and eco-climatic conditions are considered non-favourable to virus circulation.

Before the detection of WNV-L1 in the kestrel and goshawk, the Campania region was considered a low-risk area since the circulation of the virus had never been revealed by either passive or active surveillance. Nevertheless, after these cases, no further positive samples were observed for 2 years. Hereby, we describe the first clinical case of a horse infected by WNV-L1 in the Campania region and its phylogenetic relationship with the WNV-L1 strain previously found in a wild bird in the same area.

## 2 Methods

### 2.1 Case description

At the end of September 2022, an unexpected death of a 7-year-old male horse with neurological signs occurred. The owner referred the animal in the morning began to show worsening neurological symptoms, characterised by unsheathing of the penis and an inability to urinate. Within 2 h, these symptoms were followed by flaccid paresis of the rear limbs, an inability to stand, and proprioceptive deficits that progressed to recumbency. The horse died in the night. The official veterinary health authorities performed the necropsy in the field. At the opening of the body cavities, no gross lesions were observed except for an intestinal volvulus. After the outbreak was confirmed, an epidemiological investigation was conducted in order to analyse the possible source of infection.

### 2.2 Sample collection

The horse carcass underwent a post-mortem examination in the field. Heart, head, gut, spleen, and liver were entirely collected by the official veterinary services and brought to the Istituto Zooprofilattico Sperimentale del Mezzogiorno (IZSM) at refrigerated temperature. Next, in the necropsy room, the head was dissected by craniotomy, and brain samples were collected by veterinarians from the Unit of Forensic Veterinary Medicine. Furthermore, after preliminary cauterisation of their surfaces, approximately 2 cm^3^ of samples from the inner parts of each organ were taken. Official veterinary authorities also collected serum and whole blood samples from 12 horses and 3 chickens from the same stable. Horse blood was collected from the jugular vein, while chicken blood was collected from the alar vein using sterile evacuated tubes with Serum Clot Activator (Becton, Dickinson and Company, Plymouth, United Kingdom) and EDTA, following the dictates of the relevant guidelines and regulations on animal welfare.

### 2.3 Biomolecular examinations

In the Biosafety Level 3 (BLS-3) laboratory, 2 mg of each tissue sample was suspended in 1.8 mL of sterile phosphate buffer saline (PBS) in 2 ml tubes. Samples were homogenised with a 4.8-mm-diameter stainless steel bead to allow mechanical lysis using a TissueLyser (Qiagen, Hilden, Germany) for 5 min at 30 Hz and subsequently centrifuged at 1,650 × *g* for 5 min. Aliquots of 200 μL of supernatant were collected, and VetMAX Xeno Internal Positive Control RNA (Applied Biosystems, Monza, Italy) was added. Nucleic acid extraction and purification were carried out using QIAsymphony DSP Virus/Pathogen Mini Kit (Qiagen) on the QIAsymphony automated system (Qiagen) following the manufacturer’s instructions, eluted in 60 μL, and stored at −80°C until use. The eluates underwent a one-step RT-PCR assay for the simultaneous detection of West Nile virus lineages 1 and 2 according to the protocol already described by Del Almo et al. (23) and the Usutu virus as described by Cavrini et al. (24). In brief, the WNV L1–L2 reaction was carried out using the QuantiTect Probe RT-PCR Kit (Qiagen, Hilden, Germany) in a 25-μl final volume containing 5 μL of template, 1× final concentration of QuantiTect RT-PCR Master Mix, 0.4 μM for each primer, and 0.2 μM for each probe, along with 1× Xeno Liz Primer and Probe Mix and RNAse/DNase free water to reach the final volume. The thermal cycle was composed of reverse transcription at 50°C for 30 min, followed by an initial denaturation at 95°C for 15 min and 45 cycles at 95°C for 15 s and 60°C for 60 s for denaturation and annealing, respectively. USUV real-time RT-PCR was performed using TaqMan Fast Virus 1-Step Master Mix for qPCR (Thermo Fisher Scientific, Vilnius, Lithuania) in 25-μl final volume, composed of 1× final concentration for the Master Mix, 0.9 μM for each primer and 0.25 μM for the probe, 1× Xeno Liz Primer and Probe Mix, and 5 μL of template. The thermal cycle was composed of reverse transcription at 48°C for 30 min, initial denaturation at 95°C for 10 min, followed by 45 cycles of denaturation at 95°C for 15 s, and 60°C for 1 min for annealing. The reactions were carried out on a QuantStudio5 thermal cycler (Applied Biosystems) in the presence of a positive and a negative control.

Next, the RNA detected in the brain sample was fully sequenced by next-generation sequencing (NGS) technology. Briefly, RNA concentration was quantified using the Qubit RNA HS Assay Kit (Thermo Fisher Scientific, Waltham, MA, United States) and diluted to reach 100 ng final concentration. Diluted RNA was then used for library preparation, which was carried out with Illumina RNA Prep with Enrichment (L) Tagmentation (Illumina, San Diego, CA, United States), according to the manufacturer’s instructions. Finally, the enriched library quality check was performed using Tape Station 4,150 (Agilent Technologies, Santa Clara, CA, United States) and quantified using the Qubit dsDNA HS Assay Kit (Thermo Fisher Scientific). A final concentration of 10 pM was obtained and loaded onto the MiSeq platform using the v2 Reagent Cartridge, 300 cycles, and 150 bp paired-end reads. FASTQ files were generated with BaseSpace Sequence Hub (Illumina) and assembled using Geneious R9.13. The final assembly contained a total of 10,977 bp. The WNV strain sequence is available on GenBank^1^ under accession number OQ552605.

### 2.4 Phylogenetic analysis

A phylogenetic analysis was performed on the WNV L1 strain detected in the horse and on the other 18 complete coding DNA sequences downloaded from Genbank (Table 1). Sequences were obtained from *Accipiter gentilis* (*n* = 2), Blyth’s reed warbler (*n* = 1), *Culex pipiens* (*n* = 2), *Homo sapiens* (*n* = 4), horse brain (*n* = 3), house sparrow (*n* = 1), Jay (*n* = 1), and magpie (*n* = 2), as well as sequences isolated from different countries, such as France (*n* = 1), Israel (*n* = 1), Italy (*n* = 10), Morocco (*n* = 1), Senegal (*n* = 1), Spain (*n* = 1), and Turkey (*n* = 1). All sequences belonged to lineage 1. The tree was rooted by using the strain ON032498.1, belonging to lineage 2 as an outgroup.

**Table 1:** West Nile virus sequences used for phylogenetic analyses.

Strain number	Accession number	Isolation material	Host	Country	Year of isolation
West Nile virus strain Tomsk/bird/2006/A4	MN149538.1	–	Blyth’s reed warbler	Russia	2006
West Nile virus strain WNV/Turkey/HSGM136s/2018	OP617270.1	–	Human	Turkey	2018
West Nile virus strain WNV_0304h_ISR00	HM152775.1	–	Human	Israel	2000
West Nile virus strain WN Italy 1998-equine	AF404757.1	–	Equine	Italy	1998
West Nile virus strain WN 04.05 polyprotein gene	AY701413.1	Brain	Equine	Morocco	2003
West Nile virus strain France 405/04 polyprotein gene	DQ786572.1	Brain	Sparrow	France	2004
West Nile virus strain Spain/2010/H-1b	JF719069.1	Brain	Equine	Spain	2010
West Nile virus strain SN2012 241,164 polyprotein gene	ON813215.1	Mosquito Pool	Culex perfuscus	Senegal	2012
WNV 362447/20 Lin1	MW627239.1	Pool of internal organs	Goshawk	Italy	2020
West Nile virus strain 15,217	FJ483548.1	–	Magpie	Italy	2008
West Nile virus strain 15,803	FJ483549.1	–	Magpie	Italy	2008
West Nile virus isolate Italy/2008/J-242853	JF719065.1	Vero cells isolation	Jay	Italy	2008
West Nile virus strain Ita09	GU011992.2	Blood donor	Human	Italy	2009
West Nile virus strain Italy/2009/FIN	KF234080.1	Blood patient with neuroinvasive WNV	Human	Italy	2009
West Nile virus strain Italy/2021/Padova/21RS2511-3	OP009522.1	Mosquito Pool	*Culex pipiens*	Italy	2021
West Nile virus strain Italy/2022/Rovigo/22RS1560	OP609810.1	Mosquito Pool	*Culex pipiens*	Italy	2022
West Nile virus isolate ITA2022 15,935 Lin 2	ON032498.1	–	Goshawk	Italy	2022

Nucleotide sequences were aligned using the ClustalW algorithm implemented in the Molecular Evolutionary Genetics Analysis (MEGA11) software and edited manually. The maximum likelihood approach was used to infer phylogeny, and Bayesian Inference (BI) was performed using a general time reversible model with Gamma Distributed with Invariant Sites (G + I) and a gamma category count of 4. The robustness of branching patterns was tested by 500 bootstrap replications using the Hasegawa-Kishino-Yano model.

### 2.5 Serological analysis

Horse and chicken samples were transported to the IZSM laboratories under refrigerated conditions. Whole blood samples underwent real-time RT-PCR as reported above, while sera were obtained by centrifugation at 1,300 × *g* for 10 min (Centrifuge 5,810, Eppendorf, Hamburg, Germany). In line with the PNA 2020–2025, horse and chicken serum samples were tested for the presence of WNV IgM and IgG antibodies using ID Screen West Nile IgM Capture (IDvet, Grabels, France) and ID Screen West Nile Competition Multi-species (IDvet), respectively. ELISA-positive sera were then conferred to the National Reference Laboratory for exotic diseases of animals (CESME), (Istituto Zooprofilattico Sperimentale di Abruzzo e Molise, Teramo, Italy) to confirm the ELISA positivity using a virus neutralisation (VN) assay.

For this study, ethical review and approval were waived because sample collection was conducted during official routine activities in accordance with national and regional regulations and internal policy.

### 2.6 Spatial analysis

A map was produced to highlight the area where the tested-positive WNV-L1 goshawk and the dead horse of the present study were found in 2020 and 2022, respectively. Data were obtained by the Epidemiologic and Biostatistics Regional Observatory (ORSA) using qgis version 3.22 open source software^2^ with epsg projection 32,633 – wgs 84 / utm zone 33n spatial reference system.

## 3 Results

### 3.1 Epidemiological investigation

**Figure 1:**
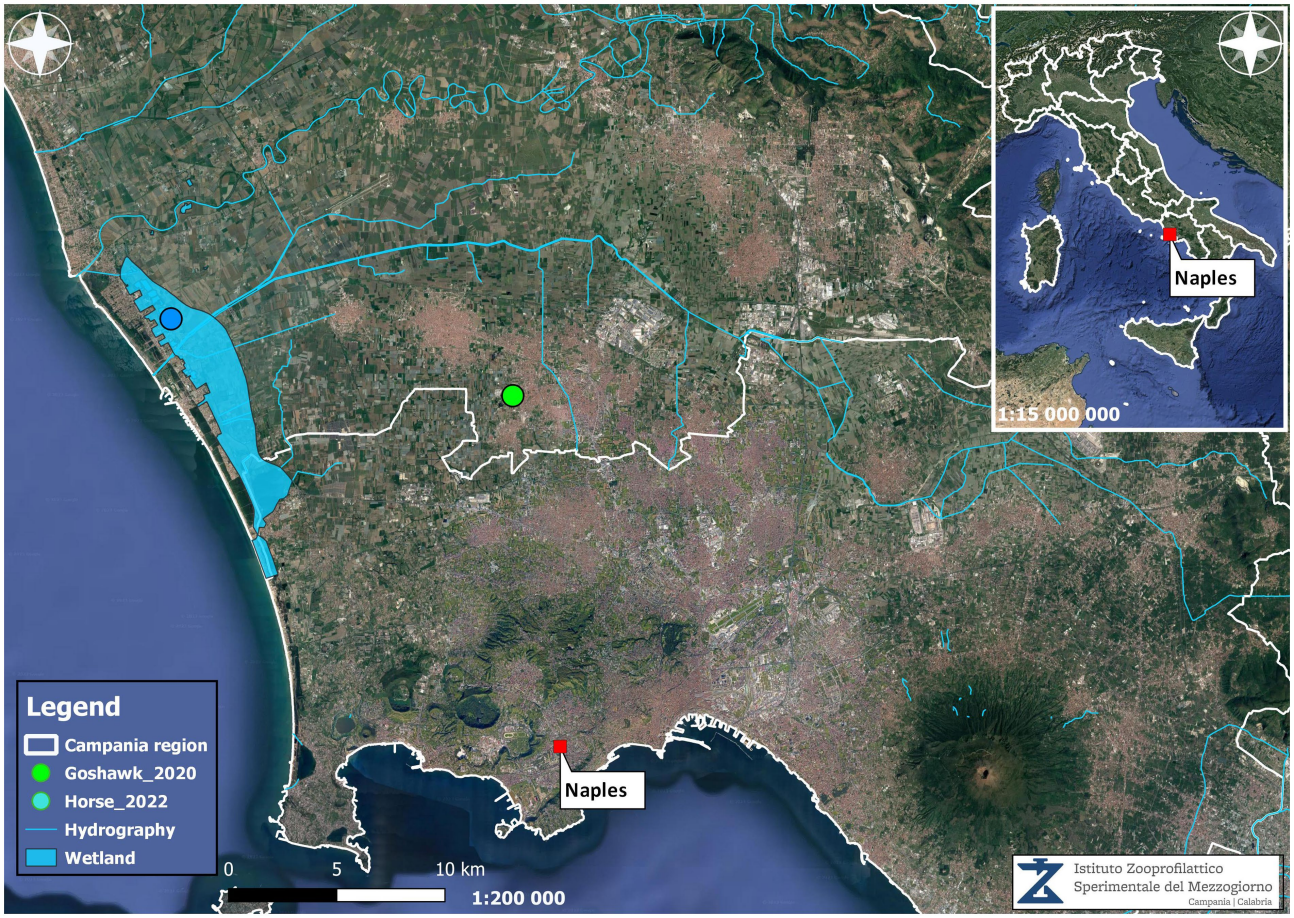
Georeferenced map showing the localisation of the tested positive horse and goshawk found in the Campania region and the environmental characteristics.

It was revealed that in the stable, there were a total of 12 horses and three chickens. No animal movements to and from the farm were reported in the previous 10 months. One of the 12 horses present on the farm, which originated from the Piedmont region of Northern Italy, was vaccinated against WNV. No signs of fever, apathy, or other symptoms were reported in the horses during the vector season. The landscape where the stable was located was characterised by wetlands that were marshlands before reclamation in the 1930s, with irrigation channels and four artificial lakes in the immediate vicinity. During spring and summer, there was an unusual abundance of mosquitoes. Periodically, the owner had performed adulticide treatments using piretroids, but no action was taken against larvae. Moreover, within 6–7 km, there is a bird-watching area widely populated by resident and migratory birds; indeed, it was a frequent observation area for wild birds, in particular ducks, corvids, hawks, and magpies. Nevertheless, no dead bird was reported in the area, except for a WNV-L1-positive goshawk found in 2020 at 17 km distance (10) (Figure 1). Furthermore, no positive mosquito pools were found by entomological surveillance, either before or after the outbreak.

### 3.2 Laboratory results

**Figure 2:**
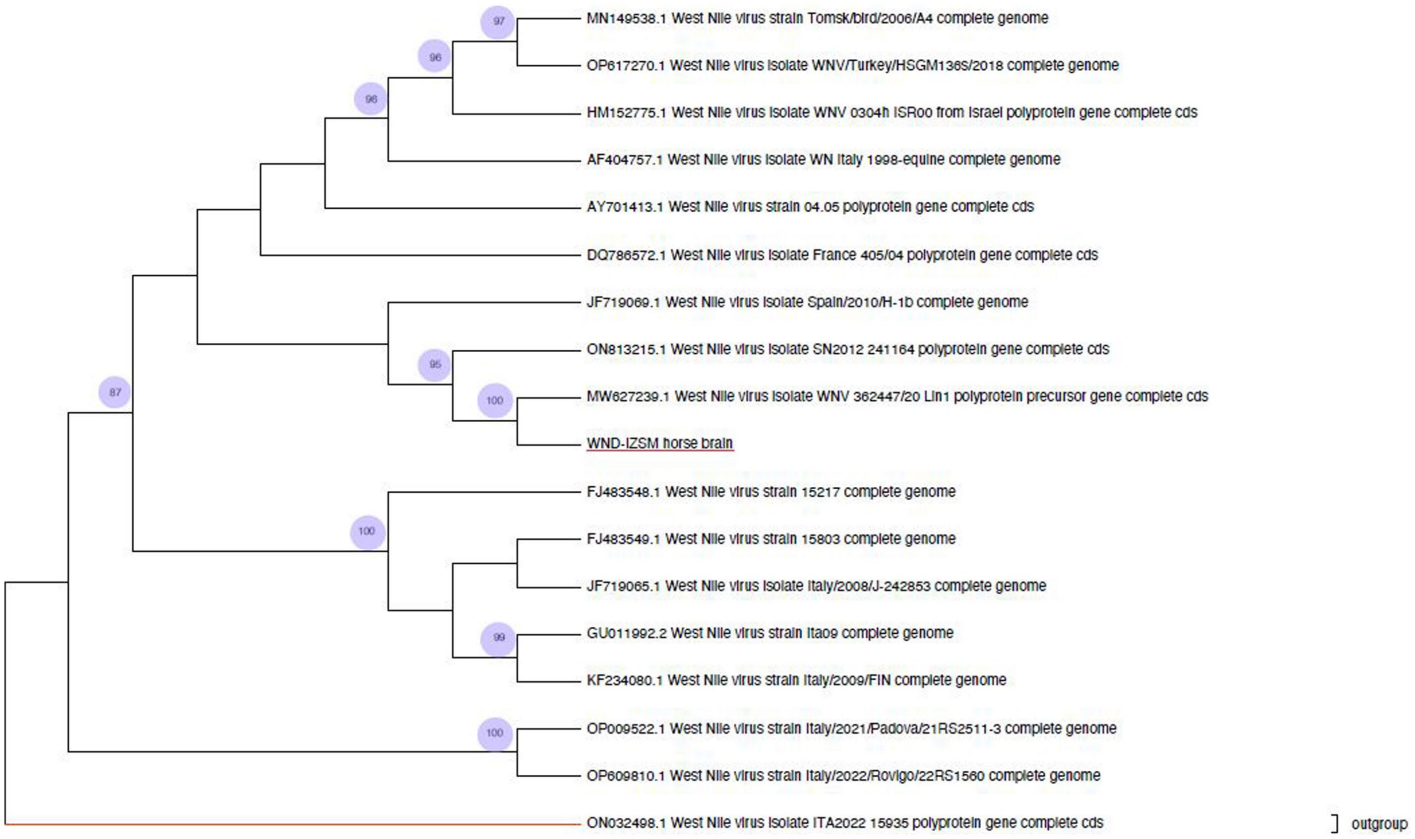
Phylogenetic analysis of West Nile virus. The present study strain obtained from the horse brain is highlighted in red.

Biomolecular tests done at IZSAM and IZSM showed that all the collected organs had WNV lineage 1. The phylogenetic analysis showed that the consensus sequence obtained by NGS (WND-IZSM horse brain, OQ552605) was similar to those of the 17 WNV L1 retrieved from GenBank (Figure 2). The values of successful bootstrapping higher than 80 are indicated at nodes. The analysis confirmed the presence of three clusters. The first cluster includes WNV-L1 strains (OP009522 and OP609810) isolated from *Culex perfucus* in Italy (Veneto Region) in 2021 and 2022. The second cluster contains five WNV-L1 strains isolated in Italy between 2008 and 2009 (FJ483548, FJ483549, JF719065, GU011992, and KF234080) from humans, magpies, and jays. The third cluster is the largest and encloses two sub-clusters with a common ancestor. One includes strains isolated (DQ786572, AY701413, AF404757, HM152775, OP617270, MN149538) from Russia, Turkey, Israel, Italy, Morocco, and France. The other includes the strain sequenced in the present study, together with the strains MW627239 isolated in the Campania region (2020), the ON813215 strain isolated in Senegal (2012), and the JF719069 strain isolated in Spain (2010). They share a sequence identity of > 98%.

Three horses and one chicken tested positive for WNV IgM and IgG, respectively. No WNV RNA was detected using real-time RT-PCR. By VN assay, the three positive horse sera showed 1:20, 1:40, and 1:320 WNV titres, while the chicken serum was 1:20.

## 4 Discussion

During the past 30 years, several outbreaks of West Nile virus have been observed in Europe, both in humans and animals, with two important peaks in 2018 and 2022 (18). Due to its geographic position, Europe, and in particular the Mediterranean basin area, is subjected to virus incursions from either the south (Africa) or the north (North Eastern Europe) through long-or short-distance migratory birds. This aspect is reflected by the high diversity of circulating strains in this area compared to other areas. Moreover, growing attention has been pointed toward the possible role of persistently infected migratory birds as a potential route of introduction and spread of WNV, as well as the USUTU virus, to the Mediterranean areas. Therefore, migratory birds could act as a virus reservoir for mosquitoes, being responsible for the establishment of a local cycle through resident bird species hosts and local vectors (25, 26). Indeed, Italy, as well as other central European countries, represents an important crossing point of migratory routes (27); thereby, they may have a crucial role as carriers of numerous microorganisms, including WNV, and parasites (1, 28, 29). As reported by Mencattelli et al. (10) in the previous outbreak in the Campania region, a goshawk and a kestrel, two species that may be considered either resident or migratory birds, could have re-introduced WNV-L1 in Italy after almost 10 years.

This study evidenced a close genetic correlation between the WNV isolated from the horse and the MW627239 goshawk strain previously isolated in the Campania region in 2020 (Figure 1), but also with the WNV-L1 Western-Mediterranean (WMed) sub-cluster, which groups the strains currently circulating in Italy and Europe.

The high nucleotide and amino acid identity of WNV-L1 strains already circulating in Europe in the past few years suggests a possible virus circulation in the Mediterranean basin area and a subsequent re-introduction through migratory birds (10). However, considering the high similarity between the goshawk MW627239 and the horse sequences, it can also be hypothesised that this strain has silently circulated for 2 years, becoming evident with the accidental infection of the horse. Indeed, WNV-L1 infections in birds might run asymptomatically or with mild symptoms (10, 30, 31). Furthermore, analysing the climatic changes over the past 40 years in the area where the horse and the raptors were found dead, a severe increase in temperature and rainfall anomalies can be observed. Indeed, from 1979 to the present day, the mean temperature increased in warmer months over the years, reflecting global warming associated with climate change. The same aspects were revealed for precipitation anomalies, with an important deviation toward wetter climatic conditions.^3^ It should also be pointed out that this geographic area is a reclaimed marshland with a significant number of waterfowl, migratory bird species, and numerous livestock establishments. Altogether, these parameters represent a possible risk of *Flavivirus* introduction (32) and may contribute to a particular abundance of certain vectors and support the potential increase and spread of vector-borne diseases. These aspects could be the basis for the persistence and overwintering of the investigated pathogen, allowing the virus to survive during the winter seasons.

In conclusion, serological and molecular active and passive surveillance conducted in Italy demonstrate that the spread of WNV is increasing, also due to climatic changes. This warrants public health measures to control vector circulation to reduce the prevalence of vector-borne diseases. Integrated surveillance of human cases and invasive and endemic mosquito species will be a milestone for the effective prevention and control of vector-borne diseases.

## Data availability statement

The datasets presented in this study can be found in online repositories. The names of the repository/repositories and accession number(s) can be found at: https://www.ncbi.nlm.nih.gov/genbank/, OQ552605.

## Ethics statement

Ethical approval was not required for the studies involving animals in accordance with the local legislation and institutional requirements because the Istituto Zooprofilattico Sperimentale del Mezzogiorno is the official laboratory designed by the Italian Ministry of Health. According to the national regulations and internal policies, ethical approval was deemed unnecessary. Written informed consent was obtained from the owners for the participation of their animals in this study.

## Author contributions

CM: Investigation, Writing – original draft. LCa: Investigation, Writing – original draft. FP: Methodology, Writing – original draft. MV: Methodology, Writing – original draft. LCo: Formal analysis, Methodology, Writing – original draft. RP: Data curation, Formal analysis, Writing – original draft. SC: Data curation, Formal analysis, Software, Writing – original draft. MA: Methodology, Writing – original draft. MG: Supervision, Writing – review & editing. FM: Supervision, Writing – review & editing. GS: Supervision, Writing – review & editing. FD’O: Conceptualization, Writing – review & editing. RP: Funding acquisition, Supervision, Writing – review & editing. GF: Conceptualization, Funding acquisition, Resources, Supervision, Writing – review & editing.

## Footnotes

^1^ http://www.ncbi.nlm.nih.gov/

^2^ https://www.qgis.org/it/site/forusers/download.html

^3^ https://www.meteoblue.com/it/climate-change/castel-volturno_italia_3179443
